# An iconic language for the graphical representation of medical concepts

**DOI:** 10.1186/1472-6947-8-16

**Published:** 2008-04-24

**Authors:** Jean-Baptiste Lamy, Catherine Duclos, Avner Bar-Hen, Patrick Ouvrard, Alain Venot

**Affiliations:** 1 Laboratoire d'Informatique Médicale et de Bioinformatique (LIM&BIO), UFR SMBH, Université Paris 13, 74 rue Marcel Cachin, 93017 Bobigny cedex, France; 2 Société de Formation Thérapeutique du Généraliste (SFTG), 233 bis rue de Tolbiac, 75013 Paris, France

## Abstract

**Background:**

Many medication errors are encountered in drug prescriptions, which would not occur if practitioners could remember the drug properties. They can refer to drug monographs to find these properties, however drug monographs are long and tedious to read during consultation. We propose a two-step approach for facilitating access to drug monographs. The first step, presented here, is the design of a graphical language, called VCM.

**Methods:**

The VCM graphical language was designed using a small number of graphical primitives and combinatory rules. VCM was evaluated over 11 volunteer general practitioners to assess if the language is easy to learn, to understand and to use. Evaluators were asked to register their VCM training time, to indicate the meaning of VCM icons and sentences, and to answer clinical questions related to randomly generated drug monograph-like documents, supplied in text or VCM format.

**Results:**

VCM can represent the various signs, diseases, physiological states, life habits, drugs and tests described in drug monographs. Grammatical rules make it possible to generate many icons by combining a small number of primitives and reusing simple icons to build more complex ones. Icons can be organized into simple sentences to express drug recommendations. Evaluation showed that VCM was learnt in 2 to 7 hours, that physicians understood 89% of the tested VCM icons, and that they answered correctly to 94% of questions using VCM (versus 88% using text, *p *= 0.003) and 1.8 times faster (*p *< 0.001).

**Conclusion:**

VCM can be learnt in a few hours and appears to be easy to read. It can now be used in a second step: the design of graphical interfaces facilitating access to drug monographs. It could also be used for broader applications, including the design of interfaces for consulting other types of medical document or medical data, or, very simply, to enrich medical texts.

## Background

The drug prescription process is complex and error-prone. Many medication errors result from incorrect prescription, leading to frequent injury, a public health problem [1-3].

The installation of computerized physician order entry (CPOE) systems within hospitals may help to decrease the frequency of such errors [4-6], although an increase in errors was recently reported after the installation of such a system [7]. For maximal efficiency, CPOEs must be linked to decision-making systems using patient data encoded in the electronic patient record [8]. This constraint restricts the use and impact of these systems, particularly for general practitioners. There is a need for improved systems for managing the risks associated with prescription drugs.

Most of these medication errors would be prevented if physicians took into account the knowledge contained in textual documents, such as drug monographs, which can accessed from their desktop. However, it is impossible for doctors to read such documents systematically when prescribing. Time is limited during consultations and it may take a long time to read these documents sufficient closely to ensure safe drug prescription. Textual documents consisting of words and numbers may not always be the most efficient way to convey the information required for the taking of rapid medical decisions [9].

The graphical approach used for road signs is recognized as an efficient way of conveying information such that drivers can take decisions rapidly. The road sign system has the following characteristics:

(1) Signs providing warnings and other useful information are based on an iconic language, which is easy to learn, combines shapes, pictograms and colors, and can be read very rapidly. This language provides the building blocks on which road signs are based.

(2) Road signs are of a fixed size and are generally located at the same place on the road to focus the driver's attention. This standardized environment makes it possible to transmit information to drivers without the need for overly complicated cognitive tasks.

We have designed a new graphical approach to make it easier for doctors to use the recommendations provided in drug monographs. This approach is similar to that used for road signs and involved two steps:

(1) The building of a graphical language, providing a set of medical icons that can be used to identify signs, diseases, physiological states, life habits, drugs and tests. The building of this language was not a simple task as the language must be both easy to learn and able to represent numerous concepts, despite the abstract nature and lack of an obvious representation of many of these concepts. In this paper – the first of two – we describe the design of VCM (*Visualisation des Connaissances Médicales*; Visualization of medical knowledge), a new iconic language based on combinatorial graphical grammar. This paper also provides the results of evaluations carried out with general practitioners, in which we assessed the comprehensibility and usability of the language.

(2) The design of a standardized environment based on interactive interfaces, using VCM language. The aim was to facilitate the cognitive tasks required for safe drug prescription. The design and preliminary evaluation of these interfaces are described in a second paper [10], illustrating the practical use of VCM.

### Graphical approaches and medicine

The presentation of information has a major impact on its accessibility and use [11]. Two types of complementary graphical approach have been proposed to facilitate access to information.

The first, information visualization, includes several techniques that have been applied to medical information [12]. These techniques, such as graphs [13,14] and table lenses [15], are efficient when used to facilitate the interpretation of quantitative medical data. They have also been applied to navigation through classifications or ontologies [16], or large amounts of text [17,18]. However, when applied to a given text, these techniques are more suitable for highlighting the structure of the text, rather than facilitating the reading of its content.

The second approach involves visualizing the content of text graphically, rather than with words or numbers, and is more suitable for facilitating the reading of the content of a long text. Humans seem to have a very high ability to remember and to recognize graphics [19]. Icons have a high cognitive impact, and can be distinguished rapidly. Graphical languages [20-22] can be used to generate icons in a consistent manner, and to build complex icons from simple primitives, facilitating their understanding. These languages are now commonly used in the everyday life. Well known examples include road signs and chemical product labeling. Other graphical languages, such as semantography [23] and O. Neurath's Isotypes [24], cover a wider field but are more complex and less widely used.

In the medical field, several graphical approaches soliciting various cognitive abilities have been developed with specific purposes. Some were proposed for monitoring changes in a particular phenomenon, such as the frequency of nosocomial infections [25] or respiratory monitoring results [26,27]. Others aim to visually present elements of the current state of a patient, such as mammography results [28], the results of a clinical trial [29], or for facilitating the comparison of antibiotic spectra [30].

Others aim to focus attention, facilitating the understanding of medical information and making that information language-independent. These approaches include sets of icons designed for use by patients [9] such as the USP set of icons [31-33]. Finally, icons can be used to facilitate memorization. In this context, Preiss *et al*. designed in 1992 a true graphical language for medical students: UVAL-MED. This language was focused on physiopathology and designed only to represent the main signs of diseases, using pictograms and a graphical grammar [34-36]. This language which does not seem to be used anywhere, cannot be used for identifying medical concepts such as diseases and drugs in a concise way.

## Methods

### VCM icon design

The design of the VCM graphical vocabulary and combinatorial grammar included three main steps: (1) selecting graphically representable attributes for diseases, signs, physiological states and life habits, drugs and tests, (2) building the vocabulary, *i.e*. the list of the graphical primitives representing these attributes and (3) defining rules for combining these primitives together and making icons.

The starting point was the representation of diseases classes, specific diseases and clinical signs. As many disease denominations include textual names (*e.g*. Alzheimer's disease), which cannot be represented graphically, we have identified a set of attributes that can help to describe and identify a given disease, such as the organ or system concerned, the etiology or the physiopathological process involved. For drugs and tests, the same problem appeared. We adopted an approach involving the systematic reuse of an entire set of icons developed for diseases and signs. We did this by representing a drug or a class of drugs in terms of their indications (*e.g*. antihypertensive drugs). Similarly, for tests, we were able to reuse the disease and sign icon system, as tests are generally used for the diagnosis or monitoring of a disease or a risk of disease.

We first considered a set of appropriate classes of graphical primitives, such as color, shape, size and pictogram, as defined by J. Bertin [37]. Each attribute was then associated with one, and only one class of graphical primitives, selected on the basis of its characteristics, such as the number of possible values (low, medium, high). Analogy (*i.e*. the use of a graphical representation that looks like the thing it represents, *e.g*. a drawing of a bacteria for a bacterial infection) and existing graphical conventions (*e.g*. male and female symbols) were used when available. A schematic style with a plain shape was used for the drawing of pictograms.

We built the graphical grammar for assembling these graphical elements into easy-to-read icons, by taking into account published results from the field of cognitive sciences or human vision [20,38-40]. Inspired by J.-G. Meunier [39], we used several methods for combining graphical primitives: juxtaposition with overlap, inclusion (one element inside the other, *e.g*. a heart pictogram inside a red square), and primitive combination (*e.g*. applying a color to a pictogram). We used the following principle to represent "is-a-kind-of" relations between concepts: If a concept A belongs to the concept class B, then the icon for B should be visible in the icon for A. For example, heart failure belongs to the class of cardiac diseases, and thus the icon for cardiac diseases should be visible in the icon for heart failure.

### VCM sentence design

A similar method was used to design VCM grammar for expressing sentences. The knowledge expressed in medical sentences in the clinical sections of drug monographs can be summarized using simple assertions involving one or a few medical concepts, such as "if disease then contraindication". A generic sentence pattern was then built to represent only these assertions, and this pattern was graphically transposed using a few geometric symbols, such as arrows.

### Evaluation methodology

The evaluation objectives were to verify that VCM could be learnt in a reasonable time, that VCM was understandable and to assess its usability.

#### Evaluator recruitment

The evaluators were standard French general practitioners recruited by the SFTG (*Société de Formation Thérapeutique du Généraliste*), an association responsible for the ongoing training of doctors throughout their careers. The evaluators attended an initial meeting at which they were briefly introduced to VCM. Then they attended a second meeting four weeks later, where the evaluation of VCM described here was performed, followed by a focus group.

#### VCM language training

Evaluators were given a training software and a paper manual to learn and experiment with the graphical language. The training software included 7 lessons for learning both VCM and the graphical interface presented in [10]. Each lesson required about 10 minutes to complete, and was composed of a short course teaching a specific part of VCM, and one or two short computer-corrected exercises. The training software was written in *HTML *with *JAVASCRIPT *and was given to the physician on a CD-ROM. Evaluators were asked to evaluate the number of hours they spent learning VCM.

#### Design of the comprehensibility evaluation

The aim of the comprehensibility evaluation was to estimate the ease with which physicians were able to remember and to understand VCM icons, and to determine which types of VCM icons were potentially difficult for physicians to learn or to understand. One hundred icons or sentences (20 current physiopathological states, 20 risks, 20 drugs, 15 tests and 25 sentences) were presented to the physicians, in a per-physician random order. Physicians were asked to indicate the meaning of the icons and sentences using natural language. The icons and sentences were selected by the evaluation designers; they ranged from the simplest to the most complex icons (in term of number of element and pictogram level of analogy) and covered various medical domains. The comprehensibility evaluation has been corrected by two persons.

Icon complexity was estimated from the number of attributes the icons used and the level of analogy of the icon's pictograms. The 75 selected icons included 24 icons with 2 attributes, 30 icons with 3 attributes and 21 icons with 4 attributes. These 75 icons included 28 with purely analogical pictograms, 20 with partly analogical pictograms, and 27 with non analogical pictograms.

#### Design of the usability evaluation

The objective of this evaluation was to determine whether physicians were able to use the information expressed in VCM. We asked physicians to find the answers to medical questions by deduction from the reading of documents (described below) and measured their response times. Each question was associated with a different document, and was asked twice: once with the document in VCM and once with text, in a per-evaluator random order. The evaluation was divided into two sequences separated by a pause of 15 minutes. Each sequence included each question once, with either the VCM or the text document. During the evaluation, the question was displayed and the evaluator was asked to click on a button to display the document, and then to click on the response. The response time was recorded by the computer, as well as the accuracy of the response (either right or wrong). Physicians were told that all questions and documents were different, although this was not actually the case. They were also told not to waste time between the document appearing on screen and giving their answer. Finally, physicians were asked to evaluate, themselves, whether they replied more rapidly and more accurately with VCM, or with text.

This evaluation was performed on Macintosh computers of equivalent performance, running MacOSX.

#### Documents and questions

We constructed fictitious drug monograph-like documents rather than using real ones, for two main reasons: (a) we wanted to avoid the possibility of physicians recognizing real drug monographs and using their memory when responding, and (b) we wanted to control the length of the documents. In addition, we also wanted that the VCM and textual versions of each document contained about the same information. Documents consisted of paragraphs corresponding to one or a few contraindications, drug interactions, cautions for use or adverse effects. There were short documents with 10 paragraphs and long documents with 30 paragraphs.

The questions asked were yes/no questions of the form "Can this drug be prescribed without precaution to a patient suffering from disease X/taking drug Y?" or "Can this drug cause adverse effect Z?". There were two types of question: questions with explicit responses, for which the document explicitly gives the response (*e.g*. the question was "Can this drug be prescribed without precaution to a patient suffering from asthma?" and the document said "This drug is contraindicated for asthma"), and questions with an implicit reply, in which the physician was required to read the whole text before deducing the response (*e.g*. for the same question, the document said nothing about asthma, and thus, implicitly, the drug could be prescribed).

There were 20 documents and questions: 10 short documents and 10 long documents, each of these two groups including 5 documents associated with a question with an explicit response, and 5 with an implicit response. The documents were built from French drug monographs. The drug monographs we used came from the French database Theriaque [41], in which drug monographs are composed of paragraphs indexed by terms.

Documents and questions were randomly generated with as few interventions on the part of the evaluation designers as possible. A paragraph database was first created by extracting all the paragraphs expressing contraindications, cautions for use, drug interactions or adverse effects from a random set of drug monographs, selected from the entire Theriaque database. Paragraphs were then automatically translated into VCM and text, using Theriaque indexing terms. Texts were manually modified to replace the name of the drug by "this drug", and simplified to be equivalent to the VCM version (as VCM is less precise than text).

Documents were created by concatenating random paragraphs from the base (without duplicates), in random order. In each document, paragraphs expressing contradictory information were manually replaced by new random ones. Questions were related to the content of a paragraph, randomly chosen in the document (for questions with an explicit response) or from the other paragraphs in the database (for questions with an implicit response).

The *PYTHON *programming language was used to design the software translating Theriaque data into VCM icons.

#### Statistical methods

For comparing the correctness of responses and the response times obtained with text and VCM, we considered three factors: evaluator, document length and question type (*i.e*. with explicit or implicit response). ANOVA was used to investigate the effect of these three factors and their interactions on response time.

Paired t-tests were carried out for comparing differences in mean response time.

For comparing the number of errors with text and VCM, Fisher's exact test was used.

Linear regression analysis was carried out to investigate the relationships between response time and percentage errors, and to take the three factors into account.

The significance threshold was set at *α *= 0.05. Data were analyzed with R software version 2.2.1 [42].

## Results

### Presentation of the VCM graphical language

#### Main principles of VCM

Diseases, signs, physiological states and life habits, drugs and tests are the medical concepts that can be represented by the VCM graphical language. The following attributes have been used to describe these concepts: anatomical and functional location, etiology, generic and specific pathological processes, timing (past or current disease, risk of future disease), the administration route (drug only) and the type of test (test only).

Each VCM icon can have a central pictogram, an external shape and, in some cases, a top right pictogram. The meaning of the icon is determined by five elements, each of which is associated with one of the attributes used to describe the medical concepts:

(i) The central pictogram may represent the anatomical and functional location of a patient state (physiological or pathological) or of the disease a drug can treat (*e.g*. a heart for cardiac treatment), possibly including disorders specific to that location (*e.g*. a heart with an ECG signal for rhythm disorders), or lifestyle or treatment properties (*e.g*. a beaker for dose).

(ii) The external shape is used to distinguish normal patient states (circle) from pathological states (square or square derivative). Shape modifiers can be applied to the square to specify the etiology or the disorders associated with a pathological state.

(iii) The color of the external shape is used to locate the patient state in the time: antecedents are shown in brown, current states in red, risks of future states in orange.

(iv) The color of the top right pictogram indicates drug treatments (green) or tests (blue)

(v) The drawing of the top right pictogram may provide additional information about drug treatments or tests (administration route for drugs, method for tests).

**Figure 1:**
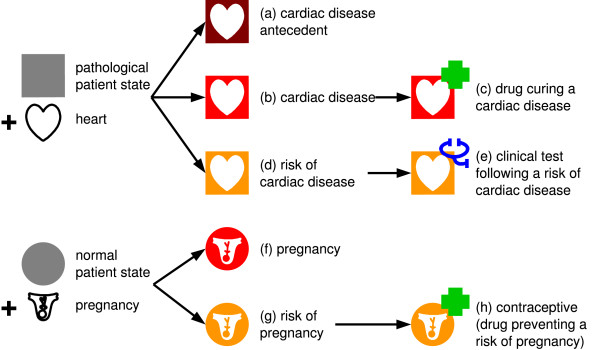
**Examples of VCM icons created by combining central pictograms, external shapes, colors and top-right pictograms**. Simple physiological or pathological states are represented by combining a color that indicates the moment at which the patient state occurs, a shape that distinguishes pathological (square) and physiological (circle) states, and a central pictogram. These icons can be reused for building drug and test icons.

Figure 1 provides examples of VCM icons and the arrows show the interdependence of various types of icons.

#### VCM icons for current diseases and signs, risks and antecedents

Anatomical and functional classes of diseases (e.g. cardiac diseases) are represented using a square as the external shape; the central pictogram indicates the anatomical and functional location involved.

**Figure 2:**
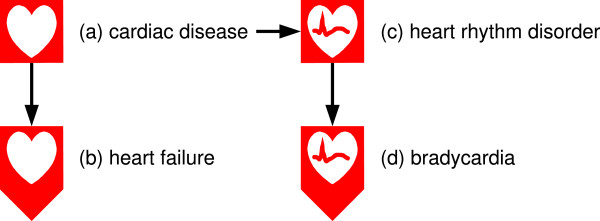
**Examples of VCM disease or sign icons**. Icon (a) simply indicates the anatomical and functional location of the disease. Icon (b) provides additional information about the general pathological mechanism, using a shape modifier, and icon (c) provides additional information about the function involved, by modifying the central pictogram. Icon (d) combines both the shape modifier and the pictogram modification.

**Figure 3:**
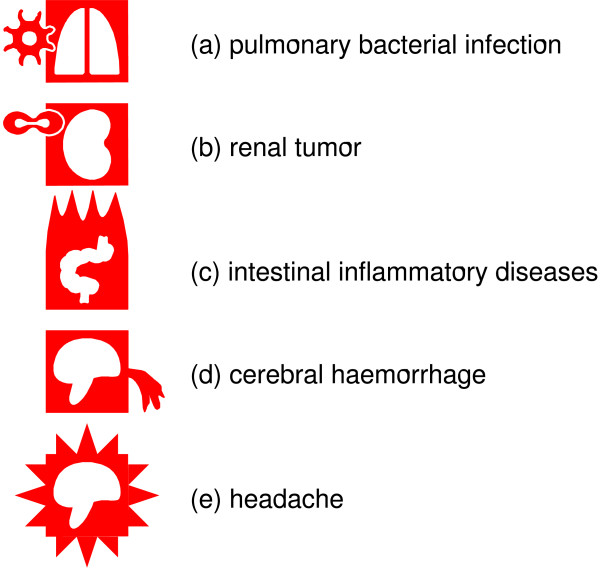
**Examples of VCM disease icons using various shape modifiers**. In icon (a) a pathological agent entering in the square from the left represents the etiology, in (b) the cell in division on the left represents a tumor process, in (c) the flames at the top of the icon represent inflammation, in (d) the bleeding on the right represents hemorrhage, and in (e) the "explosion" represents pain.

Various levels of subclasses of diseases, or single diseases, can be distinguished by indicating the pathological process. Two methods can be used to indicate the pathological process on VCM icons. For pathological processes that may occur at several anatomical and functional locations, such as the various types of infection, tumors, allergy or inflammatory process, shape modifiers are applied to the external shape. For instance, hypofunctionality (*e.g*. heart failure, Figure 2) is represented by adding a triangle to the external square shape, to suggest a downward arrow, and bacterial infection is represented by a small microbe entering the square from the left side. Figure 3 shows additional examples. The combination of possible central pictograms and external shapes yields a large number of icons from a limited set of graphical primitives.

For pathological processes occurring at a single specific anatomical and functional location, the central pictogram is modified. For example, heart rhythm disorders are specific to the heart and are represented by adding a small ECG signal in the heart (Figure 2).

Both methods can be combined in the same icon, e.g. the square with the downward arrow combined with the heart with an ECG signal indicates bradycardia.

The external shape is brown for antecedents, red for current states and orange for risks. Risks play an important role in the expression of adverse effects of drugs (see icons (a), (b) and (d) on Figure 1).

#### VCM icons for drugs

**Figure 4:**
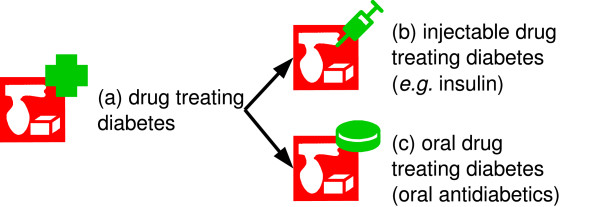
**Examples of the reuse of disease icons for building drug icons**. Icon (a) does not indicate the route of administration, whereas icons (b) and (c) do.

VCM represents the therapeutic classes of drugs using the icon of the disease they are indicated for (as given by the ATC Anatomical Therapeutical Chemical drug classification [43]). A green top-right pictogram is added to the disease icon to obtain the drug icon (see Figure 4). The top-right pictogram is a cross if the route of administration is not specified, or a tablet, a syringe or a pomade tube to indicate drugs with an oral, injectable or topical administration route, respectively.

#### VCM icons for tests

In drug monographs, tests are mostly mentioned for the follow-up of potential adverse effects of drugs. Thus, VCM represents a test using the icon of the risk it can follow up. A blue top-right pictogram is added to the risk icon to obtain the test icon. The top-right pictogram is a stethoscope, a test tube, a ray or a signal, for clinical tests, biological tests, imaging examinations and functional tests (see icon (e) on Figure 1).

#### VCM icons for physiological states and lifestyle

Patient physiological states (*e.g*. pregnancy) and characteristics (*e.g*. age classes) are represented using a circle as the external shape, and the central pictogram indicates the patient state or characteristic (*e.g*. a uterus with a baby for pregnancy, see icon (f) on Figure 1). The colors used are the same as for disease icons, with the same meaning.

#### VCM icons for drug prescription properties

Drug monographs frequently mention the various properties of the drug prescription, such as dose or dose planning. These properties are represented using a green square as the external shape, and the central pictogram indicates the property (*e.g*. the drawing of a jigger for dose). The square shape can be completed by shape modifiers to indicate a modification to be applied to the drug property (*e.g*. adding a downward arrow to the dose icon means dose reduction).

#### Building simple sentences using VCM icons

**Figure 5:**
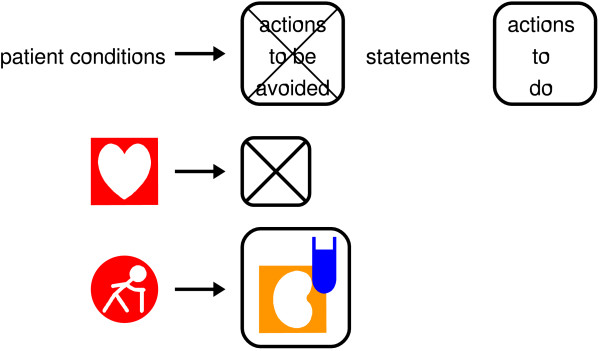
**The pattern used for VCM sentences, and two examples of sentences**. The first example means "if patient suffers from some cardiac diseases, then this drug is contraindicated (Contraindicated for some cardiac diseases)". The second means "if the patient is aged, then the physician should prescribe biological tests to monitor renal disease risks (For elderly patients, creatinine clearance tests should be prescribed)".

One or more icons can be combined with a few grammatical elements to build simple medical sentences expressing contraindications, drug interactions, cautions for use or adverse effects (Figure 5). Sentences are read from left to right and have an IF (condition) THEN (action) structure. The sentence starts with a condition of applicability (*e.g*. the icon for an elderly patient or renal failure). One to three of the following elements may then be included in the sentence, to the right: actions the physician should avoid (*e.g*. increasing the dose; when the rounded symbol is empty and struck through it means "do not prescribe the drug of the monograph"), statements (*e.g*. a risk of digestive adverse effects), and actions the physician should do (e.g. follow-up by means of laboratory testing). These elements are separated from conditions by a horizontal arrow.

AND and OR operators can be expressed in the conditions, by juxtaposing icons horizontally and vertically, respectively. In the actions that the physician should do, AND is represented by juxtaposing icons horizontally, and OR by juxtaposing icons horizontally with a vertical separation bar.

#### Current version of VCM

A complete reference manual of VCM including all the pictograms is available in Additional file 1. The current version, labelled 1.0, includes 103 central pictograms, 20 external shapes and shape modifiers, 5 colors and 8 top-right pictograms. The combination of these elements yields more than 5,000,000 potential combinations; however all of them are not of medical interest or even meaningful, *e.g*. one can create a VCM icon meaning "sleep tumor". As an indication, the various mapping we made with classifications such as ICD10 (International Classification of Diseases) or ATC led us to individualize 549 icons for diseases and 227 for treatments and follow-up procedures.

### Evaluation results

#### Evaluators and training time evaluation

We recruited 11 general practitioners (8 men and 3 women) with a mean age of 51. They attended a two-hour initial session at which VCM was presented, and then spent a mean of 4 hours (2 to 7 hours) on the VCM training software and the VCM paper manual, at home. None of the doctors withdrew from the study before the end.

#### Results of the comprehensibility evaluation

A mean of 89% of icons (95% CI: 88%–90%) and 75% (95% CI: 73%–77%) of sentences were correctly translated by the evaluators; the median were 93% and 80% respectively. Individual results ranged from 65% to 99% for icons, and from 24% to 100% for sentences. Two physicians clearly obtained poor results, both for icons and sentences. Some pictograms for psychiatric diseases were not understood by all physicians. Some other pictograms, like that for the throat, were not found very expressive and need to be improved. On the contrary, some pictograms that we felt were not particularly evident gave good results, such as the pictogram for blood, which is the combination of the three pictograms for white cells, red cells and platelets inside a blood vessel. On average, physicians give the right answer for 86% (95% CI: 81%–91%) of the icons with 2 attributes, 88% (95% CI: 84%–92%) of the icons with 3 attributes, and 95% (95% CI: 92%–98%) of the icons with 4 attributes. They gave the right answer for 92% (95% CI: 89%–95%) of the icons with purely analogical pictograms, 87% (95% CI: 82%–92%) of the icons with partly analogical pictograms, and 88% (95% CI: 84%–92%) of the icons with non analogical pictograms.

Many errors occurred in sentences using OR relations, in conditions or in actions to do.

#### Documents and questions

**Figure 6:**
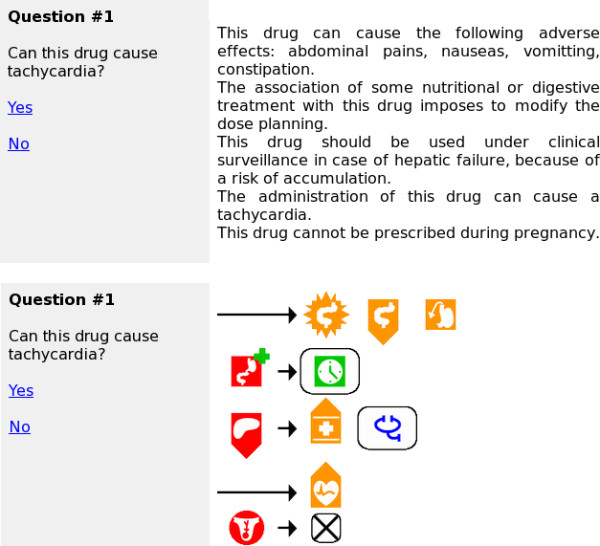
**Screenshots of the evaluation software (extracts)**. The two screenshots show the same document, expressed in text at the top, and VCM at the bottom.

We extracted 203 paragraphs from 15 randomly selected drug monographs. We constructed 20 document-question pairs. Short and long documents contained a mean of 148 and 440 words, respectively, when expressed as text, and 22 and 68 VCM icons, respectively, when expressed using VCM. Figure 6 shows screenshots of the evaluation software.

#### Results of the usability evaluation

Upon 220 responses, physicians made significantly fewer errors with VCM: 13 errors were recorded with VCM and 27 with text, respectively corresponding to 94% (95% CI: 93%–95%) and 88% (95% CI: 86%–90%) of correct answers. This difference was significant (Fisher's exact test, *p *= 0.003). The error ratio was 2.1 in favor of VCM. For text, most errors occurred with long documents, whereas for VCM, most errors occurred with long documents and questions with implicit responses. Linear regression analysis showed that there was no significant relationship between response time and the number of errors, for either VCM or text.

**Figure 7:**
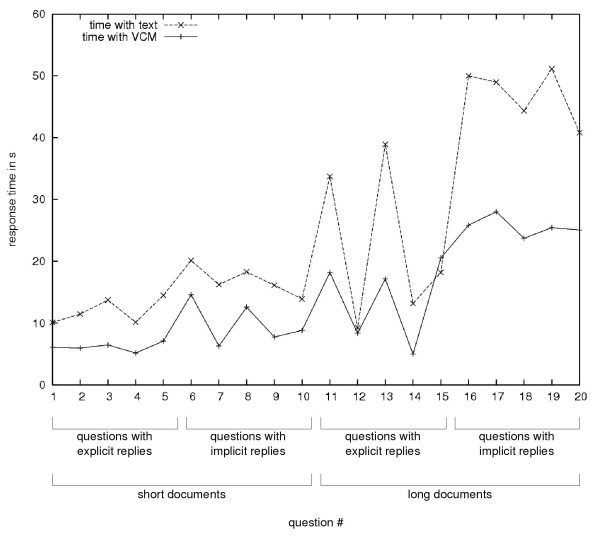
Average response time for questions answered using documents in text or VCM format.

The average response times for each question, for text and for VCM, are shown in Figure 7; response times with text and with VCM seemed to be closely linked. Physicians responded significantly more rapidly with VCM than with text (paired t-test, *p *< 0.001). The mean response time with text is 1.8 longer than with VCM; this time ratio was independent of document length or question type. All physicians answered more rapidly with VCM, with individual time ratios ranging from 1.2 to 3.4. The ANOVA analysis showed that questions associated with long documents or with implicit responses gave significantly longer response times, for both VCM and text. Consequently, the reading time with VCM icons increases with the number of icons.

When physicians were asked for their own assessment, 6 (versus 2, with the other 3 having no idea) were convinced they answered more accurately with VCM, and 10 (versus 1) were convinced they answered more rapidly with VCM than with text.

## Discussion

### VCM design

The design of VCM was based on original principles making the language easy to use and learnable in a few hours. Several obstacles have been overcome. First, it is not easy to represent the medical concepts because many of them are abstract. The graphical representation of abstract concepts was facilitated by the use of representable medical attributes well known to physicians, such as the anatomical and functional locations of diseases.

Second, the concepts to represent were very numerous, so that it would have been unrealistic to draw a specific icon per concept. Applying combinatorial rules to graphical primitives permits to solve this problem.

Third, the VCM language had to remain learnable in a few hours, so that we limited the number of primitives to be learnt, using analogy when possible in the design of pictograms, and by systematically reusing simple icons to build more complex ones.

Fourth, it must be easy to search for a given icon among a large set of icons. This searching was facilitated by the graphical representation of "is-a-kind-of" relations, *e.g*. when searching for cardiac diseases in a set of VCM icons, the physician can look for the heart disease icon, which is present in all cardiac disease icons, including the coronary disease and heart rhythm disorder icons.

"Part-of" relations are frequently defined between organs and anatomic systems, *e.g*. bronchial tubes are part of the respiratory system. However, when thinking in term of diseases, these relations translate into "is-a-kind-of" relations, *e.g*. bronchial diseases are a kind of respiratory diseases.

The number of levels of "is-a-kind-of" relations that can be represented is limited by the pictogram complexity because deeper relations would imply more complex pictograms. In practice, for a given icon component, *e.g*. a pictogram, the number of levels of "is-a-kind-of" relations is limited to 3. However, VCM icons include a central pictogram, an external shape and possibly a top right pictogram, each of them having up to 3 levels of "is-a-kind-of" relations, potentially leading to a total of 9 levels.

The medical attributes we have used for characterizing diseases are similar to the axes proposed for a formal representation of ICD10 by Héja *et al*. [44]. The choice of pictograms, shapes and colors was based on existing conventions and possible analogies, but remained somewhat arbitrary. This choice could be refined using methods involving groups of users, as proposed in previous studies [28,45]. However, these methods cannot guarantee the consistency of icons, which must rely on a graphical grammar.

VCM should not be considered a fixed language. Additional icons can be generated using the same primitives and combination rules, for a biological test aimed at the surveillance of a current disease, for example.

The structure of the sentences that we propose to build with VCM icons is very simple. This structure was derived from our analysis of the content of the drug monograph sections, and thus fits very well with the recommendations for drug use found in these documents.

We tried to avoid national or cultural specificities in the VCM language. However, the cultural independence of VCM requires rigorous evaluation and may need to be improved.

### Evaluation

The evaluation of the learning time relies on the time reported by the physicians. It can be somehow approximative, but gives an order of magnitude.

The icons and sentences used in the comprehensibility evaluation were chosen by us, because we also wanted to verify whether some potentially problematic icons were easy to learn and to understand by physicians. The results doesn't demonstrate a better comprehensibility for icons with fewer components, or icons using analogy. When we designed the study, the information needed to calculate the required number of evaluators was not available, so the number of evaluators studied was determined on the basis of practical considerations. The power of the study may therefore have been insufficient to demonstrate existing differences. Actually, this does not seem to be a real limitation, as the differences found were highly significant.

Many studies dealing with patient communication [9,33,46] have compared text with text associated with icons, using comprehension or compliance with medical recommendations as the main criterion. B. Preiss *et al*. carried out a similar analysis for the evaluation of UVAL-MED used by medical students [34], with comprehension and recall as the main criteria. However, these evaluations fail to pick up possible difficulties in understanding the graphical language: if an evaluator does not understand an icon, he will read the corresponding text, and no error will be reported. This would have been a considerable bias for a language that requires a certain training phase, like VCM.

During the usability evaluation, we could not use real medical documents, because physicians may already be familiar with these documents. We therefore used fictitious medical documents. Fictitious data are commonly used in medical evaluations; for instance Elting *et al*. [29] used "hypothetical" clinical trials with an evaluation design similar to ours. Fictitious knowledge has been less frequently used, but fictitious drug recommendations for patients have already been used by R.J. Sojourner *et al*. [33].

We used randomly generated documents rather than documents written by us, to avoid a memorization bias in the usability evaluation. In contrast, many published studies have used medical data or documents chosen or written by the evaluation designers [26,29,33,46]. In these studies, the impact of their choice remains unaddressed, as the authors may have chosen data or documents potentially favoring their graphical representation.

The evaluations were performed after a learning phase, which is required as some principles of the VCM grammar cannot be guessed, *e.g*. the use of orange for risk. In real life, physicians could progressively complete their learning of VCM using help tools such as pop-up bubbles associated with icons in medical applications.

The comprehensibility evaluation results show that many VCM icons were understood by the physicians. One of the physicians who achieved poor results (< 65%) declared having spent only two hours training. The usability evaluation results show that most physicians were able to use the information expressed with VCM more rapidly (almost twice as fast) and more accurately than textual information. This result is consistent with the physicians' own assessment.

We expected to obtain more errors with VCM than with text, because the physicians are less familiar with VCM than with text. Surprisingly, we found that the physicians made significantly fewer errors with VCM. The smaller number of errors may reflect a lower proneness to error with VCM than with text.

### Toward the use of VCM in real life

In order to be used in real life, the VCM graphical language needs to be learnt by physicians, and linked with medical components such as drug knowledge bases. This link requires a mapping between medical classifications and VCM icons.

The iconic language approach require that the physician learns the language. Even if the learning phase is not very long, it would probably be difficult to convince every physicians to spend even a few hours to learn VCM. However, VCM-based medical software could be designed to enable a progressive learning of the language. For example, textual labels describing VCM icons could be added as pop-up bubbles. These labels would allow the physician to progressively discover and learn VCM. The use of VCM during the medical studies is another option that could be considered, as other graphical approaches have been shown to help student memorization [34].

Using VCM in real life requires to map the medical classifications commonly used in medical software, such as ICD10 or ATC in VCM. VCM is not precise enough to represent all terms in these classifications, but is well-suited to represent their first levels. Lessons were learnt when translating the Theriaque thesaurus into VCM. Some therapeutic classes are named by their activities and thus their indications needed to be determined, *e.g*. diuretics are represented as anti-hypertensive in VCM, and we considered contraceptives as indicated for preventing the risk of pregnancy. Finally, a few terms would require more than one shape modifier or pictogram, whereas VCM currently allows only one of each. For instance, VCM can represent vasculitis either as an inflammatory disease or a vascular disease, but not both since inflammation and blood vessel are both shape modifiers. We are currently working on an improved version of VCM that would allow several shape modifiers per icon.

## Conclusion

Our primary objective was to build an iconic language for representing medical concepts in a graphical interface [10], to facilitate the consultation of drug monographs. The results obtained suggest that this objective has been achieved and that this language is highly usable.

The VCM language may have broader applications. It could be useful for the construction of graphical interfaces facilitating the consultation of other types of medical texts (e.g. clinical guidelines) or patient documents and the data of in the patient's electronic health record.

## Competing interests

VCM is protected by a patent, taken by University Paris 13. It is intended to be freely available for academic uses.

## Authors' contributions

J-BL, CD and AV designed the graphical language. J-BL implemented the prototype. J-BL, CD, AV and PO designed the evaluation. ABH performed the statistical analysis. J-BL, CD, AV and ABH drafted the manuscript. All authors read and approved the final manuscript.

## Pre-publication history

The pre-publication history for this paper can be accessed here:



## Supplementary Material

Additional file 1 VCM language learning and reference manual. The complete manual of the VCM language. It includes dictionaries that give the VCM icons corresponding to the main diseases, drugs therapeutic classes, laboratory tests,... This manual is an English translation of the paper manual that was given to the physician during the evaluation. Click here for file
