# Supplementary material for: An iconic language for the graphical representation of medical concepts
Source: BMC Med Inform Decis Mak. 2008 Apr 24;8:16. doi: 10.1186/1472-6947-8-16 (PMC2413217; doi:10.1186/1472-6947-8-16)
Supplement: Additional file 1 — VCM language learning and reference manual. The complete manual of the VCM language. It includes dictionaries that give the VCM icons corresponding to the main diseases, drugs therapeutic classes, laboratory tests,... This manual is an English translation of the paper manual that was given to the physician during the evaluation. [file 1472-6947-8-16-S1.pdf]

VCM language learning and reference manual

5th October 2007

Contents

1 Introduction **1**

2 The current state of the patient **2**
2.1 Physiological states, patient characteristics and patient lifestyle . . . 2
2.2 Diseases and symptoms . . . 2

3 Risks of diseases or symptoms **4**

4 Antecedents of diseases **5**

5 Treatments **5**
5.1 Non-pharmacological treatments . . . 5
5.2 Pharmacological treatments . . . 5
5.3 Pharmacological treatment properties . . . 6

6 Monitoring **6**

7 Sentences **6**
7.1 Combining several icons into a single icon . . . 7
7.2 Negations . . . 7
7.3 Actions . . . 7
7.4 AND and OR relations . . . 8
7.5 Examples . . . 8

VCM-English disease and sign dictionary **9**

VCM-English physiological state, patient characteristic and lifestyle dictionary **15**

VCM-English treatment dictionary **16**

VCM-English test dictionary **21**

Pictogram and shape lexicon **21**

1 Introduction

"VCM" is a graphical language for the visualization of medical knowledge (*Visualisation des Connaissances Médicales* in French), destined for health professionals (not rather than patients). VCM can be used to represent medical knowledge with icons. The objective is not to replace medical texts entirely by icons, but rather to combine text and icons, using icons to enable the user to localize pieces of text of interest more rapidly, *e.g.* during a medical consultation.

The current version of VCM (1.0) focuses on drug knowledge, and on SPCs (Summary of Product Characteristics, corresponding to the drug monographs) in particular. It aims to help you find answers to the following questions:

- "What are the main characterictics of this drug?"
- "Can I prescribe this drug to my patient?"
- "What should I tell my patient about this drug?"

This document presents the basics of VCM language, and includes a reference manual with an icon dictionary is included at the end.

VCM distinguishes five basic concepts, each of them is associated with a color:

- the current state of the patient, in **red**, including:
  - diseases and symptoms,
  - physiological states (*e.g.* pregnancy), patient characteristics (*e.g.* age, sex) and lifestyle (*e.g.* diet, alcohol),
- the patient risks, in **orange**, including:
  - risk of diseases or symptoms,
  - adverse effects
- the patient's antecedents, in **brown**,
- the patient's treatments, in **green**,
- the patient's medical monitoring, in **blue**.

Each medical concept is represented by an icon, consisting of a combination of colors, geometrical shapes and pictograms. However, VCM can represent only simple or general concepts. More precise concepts are represented by the more general concept that includes them.

These icons can then be used to build simple medical sentences dealing with contraindications, adverse effects, and so on.

2 The current state of the patient

The current state of the patient is represented using the **red** color. It includes diseases and symptoms, but also non-pathological states such as pregnancy.

2.1 Physiological states, patient characteristics and patient lifestyle

Physiological states, patient characteristics and patient lifestyle are non pathological states. They are represented by a red circle containing a white pictogram; the red color indicates a "current patient state", the circle indicates a "non-pathological state" and the pictogram represents the state. *E.g.*:

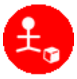
Child

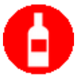
Alcohol consumption

2.2 Diseases and symptoms

VCM doesn't does not distinguish between diseases and symptoms. Both are represented as a red square containing a white pictogram; the red color indicates a "current patient state", the square indicates a "pathological state" and the pictogram indicates where the location of the disease or symptom (generally an anatomic or functional site). *E.g.*:

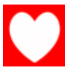
Cardiac disease

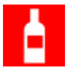
Alcoholism

The same pictogram represents both the anatomical site and the corresponding function (*e.g.* the same pictogram is used for the lung and for respiration). Diseases and symptoms may use the same pictograms as than non-pathological states, but inside these pictograms are enclosed within a square rather than a circle. Here, alcoholism is represented as a "pathological alcohol consumption".

VCM distinguishes two kinds of pathological states, making it possible to represent diseases and symptoms more precisely:

- **Pathological states that apply to several sites**, *e.g.* failures (heart failure, renal failure,...), infections (respiratory infections, urinary infections,...), cancers and tumor (pulmonary cancer, intestinal cancer,...), hemorrhages (brain hemorrhage, digestive tract hemorrhage,...). In such cases, the square is modified, the new shape providing further details concerning the type of diseases, *e.g.*:

  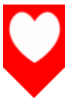
  Heart failure

- **Pathological states that apply to a single site**, *e.g.* heart rhythm disorders (heart specific), vomiting (stomach specific), epilepsy (specific to the central nervous system). In such cases, a different, more precise, pictogram is used, *e.g.*:

  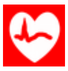 Heart rythm disorders

These two methods can be combined, *e.g.* :

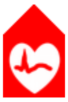 Tachycardia (increase of the heart rhythm)

VCM provides icons only for the most general, important or common diseases and symptoms. Other diseases or symptoms are represented by a more general icon, *e.g.* VCM represents “cardiac septum abnormality” as

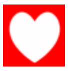 Cardiac disease

The various modifications to the square shape possible include:

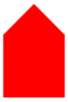 Increase / hyper-function, *e.g.*:

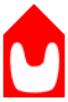 Hyperthyroidism (hyper-function of the thyroid)

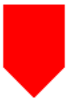 Decrease / hypo-fonction / insufficiency / failure, *e.g.*:

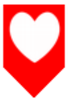 Heart failure (failure of the heart)

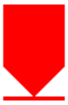 Full decrease / stop / arrest, *e.g.*:

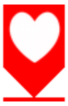 Cardiac arrest

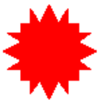 Pain, *e.g.*:

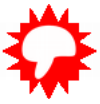 Headache (pain located in the central nervous system)

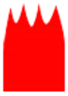 Inflammation, *e.g.*:

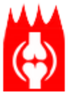 Arthritis (joint inflammation)

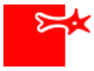 Nerve disorders, *e.g.*:

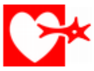 Condution disorders (nerve disorders located in the heart)

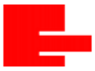 Vascular disorders, *e.g.*:.

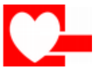 Coronary disease (vascular disorders located in the heart)

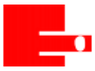 Obstructed vessel / embolism / thrombosis, *e.g*:

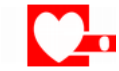 Heart infarct (obstruted vessel located in the heart)

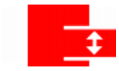 Blood pressure problems, including:

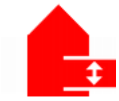 Hypertension (increase in blood pressure)

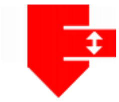 Hypotension (decrease in blood pressure)

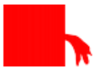 Haemorrhage, *e.g.*:

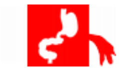 Digestive tract haemorrhage (haemorrhage located in the digestive tract)

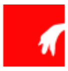 Edema, *e.g.*:

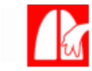 Pulmonary edema

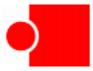 Infections, including:

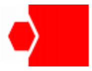 Viral infection, *e.g.*:

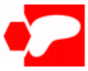 Viral hepatitis (viral infection located in the kidney)

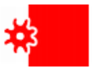 Bacterial infections, *e.g.*:

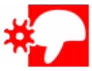 Bacterial meningitis (bacterial infection located in the central nervous system),

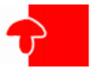 Fungus infections / mycosis, *e.g.*:

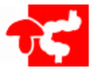 Digestive mycosis

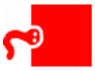 Parasitic infections, *e.g.*:

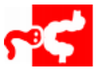 Intestinal parasitic infection

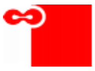 Cancers / tumors, *e.g.*:

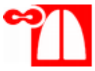 Lung cancer

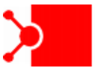 Allergy / hypersensitivity, *e.g.*:

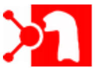 Allergic rhinitis (ear-nose-throat allergies)

3 Risks of diseases or symptoms

Risks of diseases or symptoms are represented in precisely the same way as the corresponding diseases or symptoms, but in **orange**, *e.g.*:

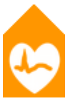 Risk of tachycardia

4 Antecedents of diseases

An antecedent of a particular disease is represented in precisely the same way as the corresponding disease, but in **brown**, *e.g.*:

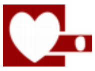 Antecedents of heart infarct

5 Treatments

Treatments are represented in **green**.

5.1 Non-pharmacological treatments

Non-pharmacological treatments are represented by a green square containing a white pictogram; the green color means "treatment", the square "non-normal state" (we consider patients following a treatment not to be in a "normal" patient state and thus use the square and not the circle for treatment), and the pictogram indicates the type of treatment:

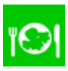 Diet

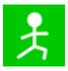 Physical activity

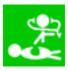 Surgical treatment

5.2 Pharmacological treatments

Drugs are represented by the icon of the disease or symptom the drug is used to treat, with a green cross superimposed on the top right-hand corner. *E.g.*:

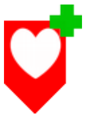 Heart failure drug / cardiotonic drug

When it is relevant, the route of administration can be specified by replacing the green cross by one of the following pictograms:

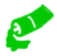 Topical drug

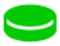 Enteral systemic route

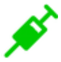 Parenteral systemic route

These pictograms can be used to distinguish between different drug classes, *e.g.*:

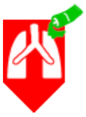 Local anti-asthmatic vs

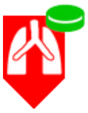 systemic anti-asthmatic

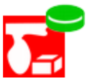 Oral anti-diabetic vs

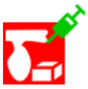 insule treatment

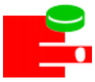 Vitamin K inhibitors vs

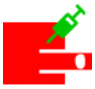 heparin

VCM provides icons only for the most general, common or important therapeutical classes. More specific classes or drugs indicated by ICD or brand name are represented by the icon for the corresponding therapeutic class. *E.g.* for propranolol:

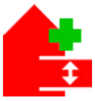 Anti-hypertensive drug

5.3 Pharmacological treatment properties

The properties of drug treatments are represented by a green square containing a white pictogram; the square may be replaced by the increase, decrease or stop shapes. Events associated with drug administration (*e.g.* overdose) are represented by a red square, and risks of such events, by an orange square.

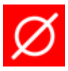 Absence of effect / treatment failure

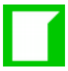 Change dose

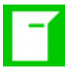 High dose

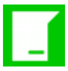 Low dose

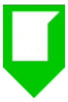 Decrease dose

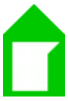 Increase dose

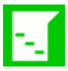 Taper dose / decrease the dose slowly

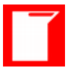 Overdose

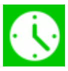 Change dose schedule

6 Monitoring

Monitoring is indicated by the icon of the risk which is controlled, with a **blue** pictogram added at the top right-hand corner. The pictogram indicates the type of test associated with this monitoring:

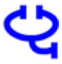 Clinical test

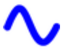 Functional test

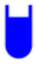 Biological test

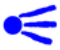 Imaging test

E.g.:

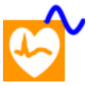 ECG (functional surveillance of the heart rhythm)

7 Sentences

VCM icons can be combined to build graphical sentences. These sentences can represent sentences extracted from SPCs or drug monographs. The drug described by the SPCs is not explicitely stated in the sentence. So, VCM sentences say *e.g.* "contraindicated for asthma" and not "drug XXX is contra-indicated for asthma".

In VCM, all sentences follows this pattern:

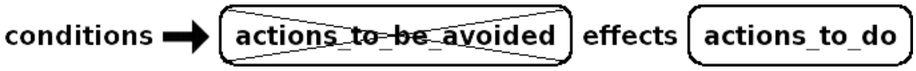

"conditions" are the conditions needed for the rest of the sentence to apply, *e.g.* a sentence may apply only to patients suffering from a given disease. "effects" indicates the effects or properties of the drug described by the SPC (*e.g.* adverse effects). "actions_to_do" and "actions_to_be_avoided" indicate the actions that should be done or that should be avoided.

The conditions are followed by an arrow, all actions are framed and the actions to be avoided are crossed out. The statements are represented "as-is". All the four elements are optional, and if one is not present, it is hidden. *E.g.* if a sentence has no condition, the "conditions" block and the arrow are hidden.

In some situations, several VCM sentences are required to represented a single safe practice. In this case, the sentences are placed on the same line, and separated by a semi-colon.

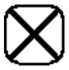 ("actions to be avoided" with no icon inside) means "do not prescribe the drug described by the SPC". This symbol is frequently used for contraindications.

7.1 Combining several icons into a single icon

VCM can combine several icons, representing them by a more general icon, in order to reduce the length of a sentence. *E.g.* "heart rhythm disorders" and "angor" can be combined and represented as "several cardiac diseases". In this case, a shadow is added behind the icon, to indicate the superimposition of several icons. *E.g.*

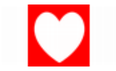 Cardiac disease

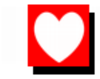 Several cardiac diseases

7.2 Negations

The icon is negated by crossing it out, *e.g.*:

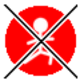

= Sedentary patient (no physical activity)

7.3 Actions

Actions the physician should undertake or avoid may be of different types:

Prescribing a treatment: the icon of the treatment of drug to be prescribed is used, *e.g.*:

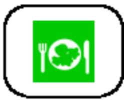 = The prescription of a diet is recommended

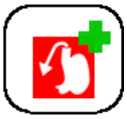 = The prescription of an antiemetic is recommended

Modifying and stopping the current treatment: the icon corresponding to the modification is used, *e.g.*:

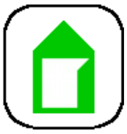 = An increase in dose is recommended

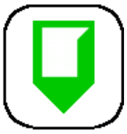 = A decrease in dose is recommended

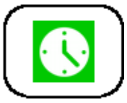 = A change in dose schedule is recommended

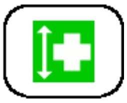 = The treatment should be stopped

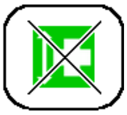 = The treatment should not be stopped

Carrying out or prescribing monitoring, *e.g.*:

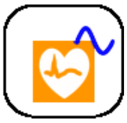

= An ECG should be performed

7.4 AND and OR relations

Logical AND and OR relations are represented differently for conditions and actions to be avoided or done:

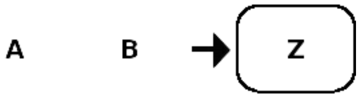

= If A and B,...

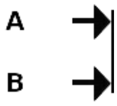

= If A or B,...

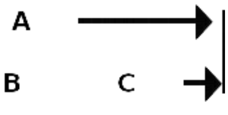

= If A or (B and C),...

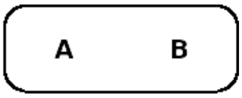

= A and B should be carried out

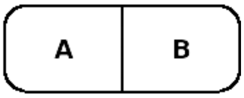

= A or B should be carried out

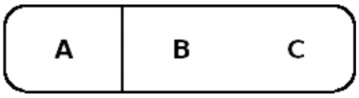

= A or (B and C) should be carried out

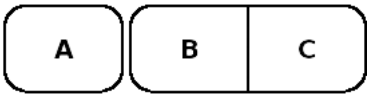

= A and (B or C) should be carried out

7.5 Examples

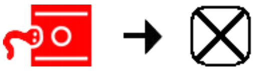

= Contraindicated for malaria

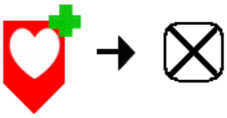

= Contraindicated for patients taking drugs for heart failure

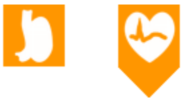

= Adverse effects: digestive problems and bradycardia

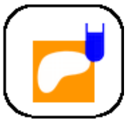

= Hepatic laboratory tests are recommended

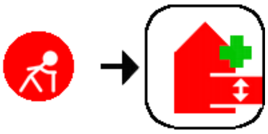

= For elderly patients, prescribe an anti-hypertensive drug

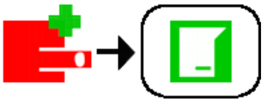

= Safe practice with anti-thrombotic drugs: the dose should be reduced

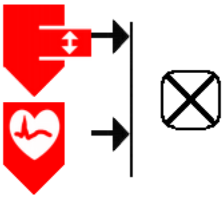

= Contraindicated for hypotension or bradycardia

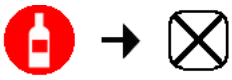

= Contraindicated with alcohol

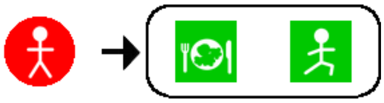

= For adult, prescribe a diet and physical activity

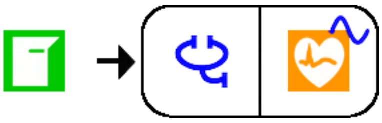

= If the dose is high, a clinical and an electrocardiographic monitoring should be carried out

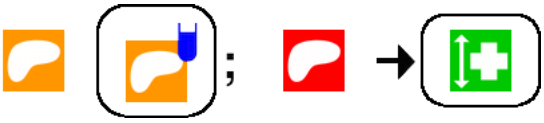

= Risk of hepatic problems, requiring hepatic monitoring; if an hepatic problem occurs, stop the treatment.

VCM-English disease and sign dictionary

This dictionary gives the VCM icons for the main diseases and signs; however, it is absolutely not exhaustive.

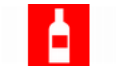

Alcoholism

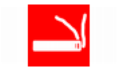

Smoking

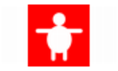

Obesity

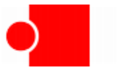

Infection

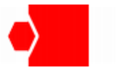

Viral infection

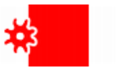

Bacterial infection

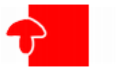

Fungal infection

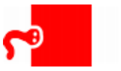

Parasitic infection

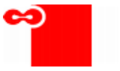

Cancer, tumor

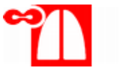

Lung cancer

etc

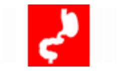

Digestive tract diseases

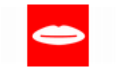

Buccal diseases

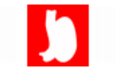

Esophageal and stomachal diseases

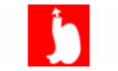

Gastroesophageal reflux disease (GERD)

- Nausea / vomiting
- Peptic ulcer
- Diseases of the small intestine
  - Intestinal fungal infections
  - Diarrhoea (increase in intestinal transit)
  - Constipation (decrease in intestinal transit)
  - Intestinal obstruction (cessation of the intestinal transit)
  - Inflammatory bowel disease
  - Digestive bleeding
- Diseases of the large intestine, the rectum and the anus
- Hepatic diseases
  - Viral hepatitis
  - Hepatic failure
- Disorders of the biliary tract
  - Cholelithiasis

Eating disorders

- Anorexia nervosa (decrease in food intake)

Endocrine and metabolic diseases

- Enzymatic disorders
- Thyroid diseases
  - Hyperthyroidism
  - Hypothyroidism

- Diabetes
- Hypoglycemia
- Dyslipidemia

Cardiac diseases

- Coronary artery disease (vascular disease located in the heart)
  - Coronary thrombosis (obstructed vessel located in the heart)
- Heart rhythm disorders
  - Tachycardia (increase in cardiac rhythm)
  - Bradycardia (decrease in cardiac rhythm)
- Cardiac conduction disorders (nerve disorder located in the heart)
- Heart failure
- Cardiac arrest

Vascular diseases

- Blood pressure diseases
  - Hypertension
  - Hypotension

Blood diseases

- Hydro-electrolytic disorders
- Red cell diseases
  - Malaria
  - Anemia
- Platelet and coagulation disorders

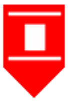 Hemophilia

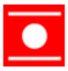 White cells and immune system disorders

- 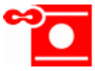 Leukemia
- 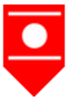 Immunodeficiency (*e.g.* neutropenia,...)

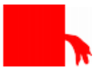 Hemorrhage

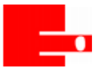 Thrombosis

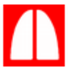 Respiratory diseases

- 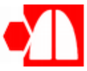 Viral pneumopathy
- 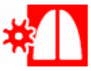 Bacterial pneumopathy
- 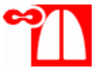 Lung cancer
- 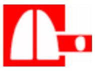 Pulmonary thromboembolism
- 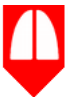 Respiratory failure
- 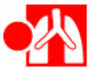 Bronchitis
- 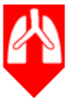 Asthma, chronic obstructive pulmonary disease (COPD)
- 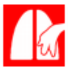 Pulmonary edema

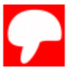 Central nervous system diseases

- 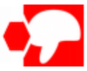 Viral meningitis
- 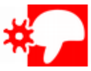 Bacterial meningitis
- 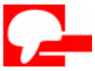 Cerebrovascular diseases
- 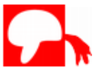 Cerebral haemorrhage
- 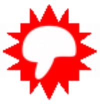 Headache
- 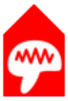 Epilepsy (increase in cerebral activity)
- 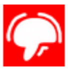 Parkinson's disease (central nervous system + tremor)

- Alzheimer's disease (central nervous system + "memory holes")

Psychiatric and behavioural diseases

- Mood disorders
  - Manic disorder (increase in mood)
  - Depression (decrease in mood)
- Sleep disorders
  - Sleepiness, hypersomnia (increase in sleep)
  - Insomnia (decrease in sleep)
- Drug dependence
- Vertigo
- Anxiety
- Asthenia

Bone diseases

- Fracture
- Osteoporosis (bone with holes)
- Paget's disease (reshaped bone)

Joint disorders

- Gout, joint pain
- Arthritis (inflammation of a joint)
- Arthrosis (functional decrease of a joint)

Muscle diseases

- Myalgia

- Myasthenia

- Eye diseases
  - Glaucoma
  - Vision deficit (including myopia, diplopia,...)
  - Blindness
  - Optic nerve disorders

- Ear diseases
  - Infectious otitis
  - Deafness (decrease in hearing)

- Nose and throat diseases
  - Infectious rhinitis
  - Allergic rhinitis
  - Cough

- Skin diseases
  - Cutaneous inflammation
  - Photosensitivation (skin + sun)
  - Psoriasis (skin + red plaques)
  - Acne (skin + pimples)
  - Rash, pruritis, erythema,...
  - Hair loss

- Renal disease
  - Renal cancer

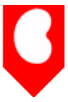
Renal failure

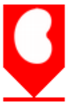
Terminal renal failure (cessation of renal function)

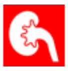
Urinary tract diseases

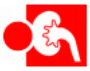
Urinary tract infection

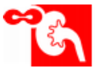
Urinary bladder cancer

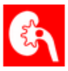
Urinary lithiasis

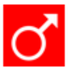
Male reproductive system disease

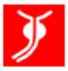
Prostate diseases

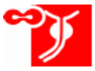
Prostate cancer

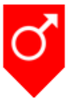
Impotence

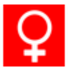
Female reproductive system disease

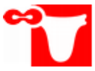
Uterine cancer

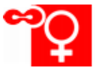
Ovary cancer

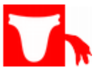
Abnormal uterine bleeding

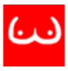
Breast diseases

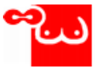
Breast cancer

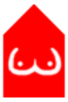
Gynecomastia

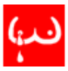
Breastfeeding disorders

VCM-English physiological state, patient characteristic and lifestyle dictionary

VCM distinguishes four age classes:

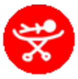
Infant

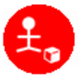
Child

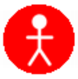
Adult

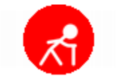 Elderly patient

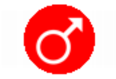 Male

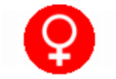 Female

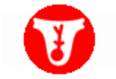 Pregnancy

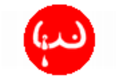 Breastfeeding

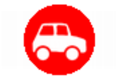 Car driving and machine usage

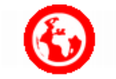 Travel

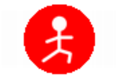 Physical activity

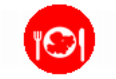 Diet and food intake

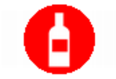 Alcohol

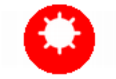 Sun exposure

VCM-English treatment dictionary

This dictionary gives the VCM icons for the main treatments; however, it is absolutely not exhaustive.

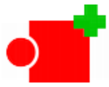 Anti-infection drugs

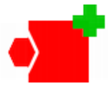 Antiviral drugs

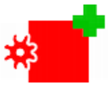 Antibiotics

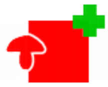 Antimycotics

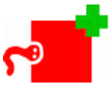 Antiparasitic drugs

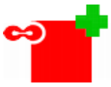 Antineoplastic drugs

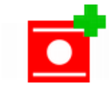 Drugs for immune diseases (including immuno-suppressive drugs)

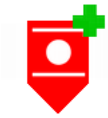 Immunotherapy

- Analgesic
- Anti-inflammatory drugs
- Digestive drugs
  - Stomachal drugs
    - Antiacids
    - Anti-ulcer drugs
    - Antiemetics
  - Intestine drugs
    - Drugs for intestinal infections
    - Anti-diarrhoea drugs
    - Laxatives
  - Drugs for diabetes
    - Insulin
    - Oral drugs for diabetes
  - Vitamins, food supplements
- Cardiovascular drugs
  - Cardiotonics
  - Anti-anginal agents
  - Antiarrhythmics

- 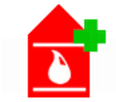 Lipid-lowering drugs
- 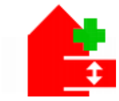 Anti-hypertensive drugs

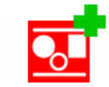 Hematologic drugs

- 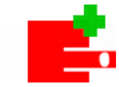 Anti-coagulants
  - 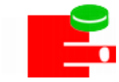 Vitamin K inhibitors
  - 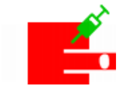 Heparin and derivatives
- 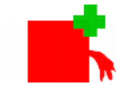 Antihaemophilia drugs
- 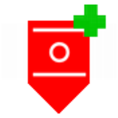 Anti-anemic drugs
- 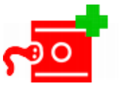 Anti-malaria agents

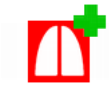 Drugs for the respiratory tract

- 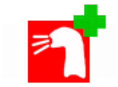 Antitussives
- 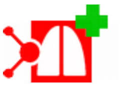 Drugs for pulmonary allergy
- 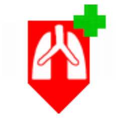 Anti-asthma drugs
  - 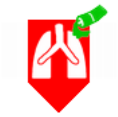 Topical anti-athma drugs
  - 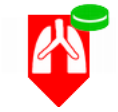 Oral systemic anti-asthma drugs

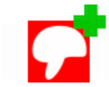 Drugs for nervous system diseases

- 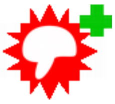 Anti-migraine drugs
- 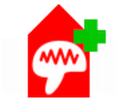 Anti-epileptic drugs

- Drugs for Parkinson’s disease
- Drugs for psychiatric diseases
  - Psycholeptic drugs
    - Anxiolytics
    - Sedatives and hypnotic agents
  - Psychoanaleptic drugs
    - Antidepressants
- Drugs for bone disorders
- Drugs for joint disorders
  - Antigout drugs
- Drugs for muscle disorders
  - Muscle relaxants
- Ophthalmic agents
  - Topical drugs for eye infections
  - Anti-glaucoma drugs
- Otological agents
  - Topical drugs for ear infections
- Dermatologic agents

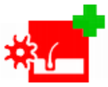
Antiseptics, disinfectants

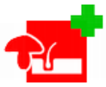
Antifungal agents

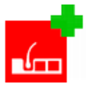
Antipsoriatic drugs

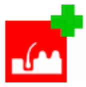
Anti-acne drugs

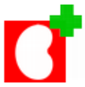
Urological agents

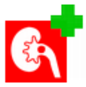
Urinary concrement solvents

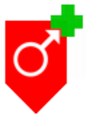
Drugs used for erectile dysfunction

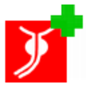
Drugs for prostate disorders

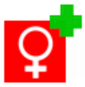
Gynecologic agents

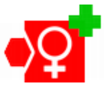
Drugs for gynecologic infections

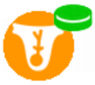
Oral contraceptives (*i.e.* drugs eliminating the risk of pregnancy)

Concepts associated with drug treatments

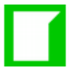
Dose

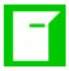
High dose

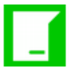
Low dose

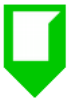
Reduce the dose

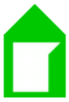
Increase the dose

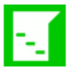
Need for a weaning (dose tapering) / reduce the dose slowly

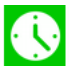
Dose schedule

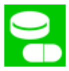
Therapeutic formulation

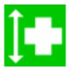
Stop the treatment

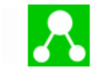 Notable excipient

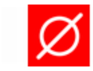 The treatment has no effect / treatment failure

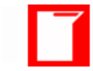 Overdose

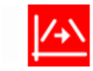 May change some test results

VCM-English test dictionary

This dictionary gives the VCM icons for the main tests; however, it is absolutely not exhaustive.

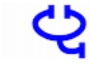 Clinical surveillance

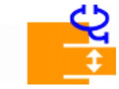 Blood pressure surveillance

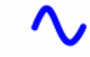 Functional surveillance

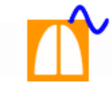 Lung function tests

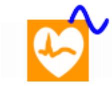 ECG

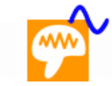 EEG

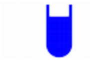 Laboratory tests

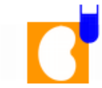 Laboratory tests for renal activity (clearance)

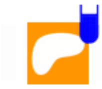 Laboratory tests for hepatic activity (transaminases)

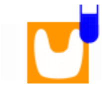 Laboratory tests for thyroid activity

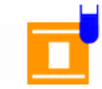 Blood coagulation tests (INR, prothrombin time,...)

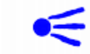 Imaging

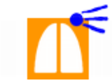 Chest X ray

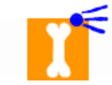 Bone X ray

Pictogram and shape lexicon

Shapes

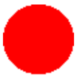 normal, physiological state

- Hyperfunction, increase, increased
- Hypofunction, decrease, decreased
  - Arrest
- Inflammation
- Pain
- Infection
  - Bacterial infection
  - Viral infection
  - Fungal infection
  - Parasitic infection
- Tumor, cancer
- Allergy
- Vascular
  - Blood pressure
    - Hypertension
    - Hypotension
  - Edema
  - Haemorrhage
  - Obstructed vessel, thrombosis
- Nervous trouble

Anatomico-functional location

- Temperature
- Lung, respiration
  - Bronchus

- Heart
  - Cardiac rhythm
- Blood
  - Red cells
  - White cells, immunity
  - Platelets, coagulation
  - Plasma, water balance
    - Glycemia
    - Lipidemia
    - Drug plasmatic concentration
- Genital system
  - Male genital system
    - Prostate
  - Female genital system
    - Uterus
    - Breast
      - Breastfeeding
- Pregnancy
  - Pregnancy, first trimester
  - Pregnancy, second trimester
  - Pregnancy, third trimester
- Kidney, urinary system
  - Bladder, urinary tract
    - Urinary lithiasis
- Diet and food intake
- Mouth
  - Throat, nose
    - Cough

- Digestive tract, digestion
  - Stomach
    - Vomitting, nausea
    - Gastroesophageal reflux disease (GERD)
    - Ulcer
  - Small intestine
  - Large intestine, rectum
  - Liver
    - Gallbladder, biliary tract
      - Cholelithiasis
- Gland, endocrine system
  - Thyroïd
  - Enzymatic disorders
  - Diabetes
- Brain, central nervous system
  - Cerebral activity
  - Alzheimer disease
  - Parkinson disease, Parkinson syndrom
- Behaviour, cerebral function
  - Sleep
  - Mood
  - Asthenia
  - Anxiety
  - Vertigo
  - Drug dependence
- Eye, vision
  - Glaucoma
- Ear, hearing
- Skin

Photosensibilisation

Psoriasis

Rash, pruritus, erythema

Acnea

Hair loss

Bones

Fracture

Osteoporosis

Paget disease

Join

Muscle, connective tissue

Patient characteristics and lifestyle

Age: infant

Age : child

Age : adult

Age : elderly

Sun exposure

Car-driving and machine usage

Geography

Sport

Alimentation

Alcohol

Smoking

Overweight, obesity

Treatment

Stop the treatment

Drug, monotherapy

Surgery

- General anaesthesia
- Dose
  - Low dose
  - High dose
    - Overdose
  - Weaning, withdrawal, progressive dose reduction
- Dose schedule, chronopharmacology

Route of administration

- Oral
- Topical
- Parenteral

Test

- Clinical test
- Biological test
- Imaging test
- Functional test

Total : 120 pictograms and shapes.
